# Gut Microbiota in Heart Failure with Preserved Ejection Fraction: Clinical Phenotyping and Therapeutic Opportunities

**DOI:** 10.3390/biomedicines14092123

**Published:** 2026-09-19

**Authors:** Goran Loncar, Milovan Bojic, Jelena Repac, Natasa Cvetinovic, Marija Rakic, Tamara Nedeljkovic, Tanja Lunic, Danilo Obradovic, Stephan von Haehling, Mitja Lainscak, Petar Otasevic, Masa Petrovic, Nikola Blagojevic, Danka Vukasinovic, Bojan Bozic, Biljana Bozic Nedeljkovic

**Affiliations:** 1 Dedinje Cardiovascular Institute, 11000 Belgrade, Serbia; loncar_goran@yahoo.com (G.L.); dedinje@ikvbd.com (M.B.); ncvetinovic@gmail.com (N.C.); tnedeljkovic006@gmail.com (T.N.); potasevic@yahoo.com (P.O.); 5rovicmasa@gmail.com (M.P.); bbozic@bio.bg.ac.rs (B.B.); 2 Faculty of Medicine, University of Belgrade, 11000 Belgrade, Serbia; 3 Institute of Physiology and Biochemistry “Ivan Djaja”, Faculty of Biology, University of Belgrade, 11000 Belgrade, Serbia; marija.mandic@bio.bg.ac.rs (M.R.); tanja.lunic@bio.bg.ac.rs (T.L.); biljana@bio.bg.ac.rs (B.B.N.); 4 Department for Internal Medicine and Cardiology, Heart Centre Dresden, Faculty of Medicine and University Hospital Carl Gustav Carus, TUD Dresden University of Technology, 01307 Dresden, Germany; daniloobradovic2@gmail.com; 5 Department of Cardiology and Pneumology, University Medical Center Göttingen, 37075 Göttingen, Germany; stephan.von.haehling@med.uni-goettingen.de; 6 DZHK (German Centre for Cardiovascular Research), Partner Site Lower Saxony, 37075 Göttingen, Germany; 7 General Hospital Murska Sobota, 9000 Murska Sobota, Slovenia; mitja.lainscak@guest.arnes.si; 8 Faculty of Medicine, University of Ljubljana, 1000 Ljubljana, Slovenia; 9 Center for Heart Failure, Cardiology Department, University Clinical Center “Dr. Dragisa Misovic-Dedinje”, 11000 Belgrade, Serbia; n.blagojevic0205@gmail.com; 10 Institute of Hygiene and Medical Ecology, Faculty of Medicine, University of Belgrade, 11000 Belgrade, Serbia; danka.vukasinovic@med.bg.ac.rs

**Keywords:** heart failure with preserved ejection fraction, gut microbiota, clinical phenotyping, gut–heart axis, functional prediction

## Abstract

**Background/Objectives**: In heart failure with preserved ejection fraction (HFpEF), the gut–heart axis has emerged as a potential contributor to inflammation and cardiometabolic stress. We characterised gut microbiota composition, diversity, and predicted metabolic potential in HFpEF to assess their associations with disease status and clinical heterogeneity. **Methods and Results**: Faecal samples from 30 HFpEF patients and 28 healthy controls were profiled by means of bacterial ribosomal RNA gene sequencing. The groups were comparable in age (74 years in both groups) and sex (female: 60% vs. 60.7%). HFpEF patients exhibited lower community evenness (*p* = 0.04) and a trend toward lower diversity (*p* = 0.08), but no detectable shift in global community structure. The tested clinical and demographic variables explained only a modest proportion of the observed variation in community structure, with substantial residual variation across the beta-diversity analyses. No taxa were differentially abundant between groups at the predefined thresholds. Among clinical stratifications, New York Heart Association (NYHA) class showed the most consistent family-level associations, including stepwise depletion of *Lachnospiraceae*, *Butyricicoccaceae*, and *Ruminococcaceae* and enrichment of *Akkermansiaceae*; *Enterobacteriaceae* peaked in milder disease. These severity-associated taxonomic trajectories were accompanied by predicted functional shifts consistent with microbial adaptation to increasing metabolic constraints, including enrichment of stress-adaptation traits and, with increasing functional limitation, depletion of core metabolic and fermentative pathways and relative enrichment of oxidative stress-response pathways. **Conclusions**: HFpEF is associated with subtle gut microbiota alterations that appear to reflect severity- and context-dependent ecological reorganisation rather than uniform dysbiosis. These findings support severity-informed approaches targeting microbial metabolic function and ecological context rather than broad compositional changes.

## 1. Introduction

Heart failure (HF) is a complex syndrome resulting from structural and functional cardiac abnormalities, leading to fatigue, fluid retention, and dyspnoea [1]. Based on left ventricular ejection fraction (LVEF), HF is classified as reduced (HFrEF), mildly reduced (HFmrEF), or preserved (HFpEF) [2]. HFpEF accounts for over half of HF cases and is associated with high mortality, frequent hospitalisations, and significant chronic morbidity. Unlike HFrEF, which is often triggered by direct myocardial injury, HFpEF develops mainly in the context of comorbidities such as hypertension, obesity, diabetes mellitus (DM), and renal dysfunction [3]. Despite its prevalence, HFpEF remains difficult to diagnose and treat, underscoring the need for mechanistic insights and new therapeutic strategies.

Evidence indicates that HFpEF is a systemic disease driven by chronic low-grade inflammation, endothelial dysfunction, oxidative stress, and immune dysregulation [4]. The gut–heart axis has emerged as a key contributor, with the gut microbiota (GM) implicated in chronic, autoimmune, and cardiovascular diseases [5]. Altered GM composition can impair intestinal barrier integrity, allowing translocation of microbial products and endotoxins into the circulation, sustaining systemic inflammation [6,7]. In HFpEF, these changes may exacerbate endothelial dysfunction and oxidative stress, promoting disease progression [8].

Recent studies have identified alterations in GM in HFpEF, including compositional changes, reduced functional potential, and depletion of short-chain fatty acid (SCFA)-producing taxa [7,9]. However, clinical data remain limited and heterogeneous, largely restricted to case–control comparisons, and provide an incomplete understanding of GM variation across disease severity and phenotypic subgroups.

Despite increasing evidence linking the GM to cardiovascular health, its role in HFpEF remains poorly understood. We hypothesised that patients with HFpEF exhibit distinct gut microbial diversity, composition, and functional potential compared with healthy controls, and that these features vary according to disease severity and clinical phenotype. To address this gap, we characterised the GM in a clinically well-phenotyped HFpEF cohort, integrating demographic, clinical, metabolic, and lifestyle data. We further examined whether microbial taxonomic and predicted functional profiles were associated with disease severity and relevant patient characteristics.

## 2. Materials and Methods

A brief overview of the study design and analytical procedures is provided below, while detailed experimental protocols are described in the Supplementary Information (Supplementary Methods).

### 2.1. Study Population and Sample Collection

Faecal deoxyribonucleic acid (DNA) was analysed from 58 individuals (30 HFpEF patients, 28 healthy controls) after excluding 2 controls due to technical issues and divergent sequencing. Clinical metadata, including demographic, anthropometric, echocardiographic, laboratory, and functional data, were collected. Participants were stratified by body mass index (BMI, lower BMI < 25 kg/m^2^, higher BMI ≥ 25 kg/m^2^) and New York Heart Association (NYHA) class (I–II as mild, III–IV as severe). Study inclusion and exclusion criteria are detailed in Supplementary Methods Table S1. Demographic, metabolic, and lifestyle data (including BMI, BSA, smoking status, SGLT2 inhibitor use, breakfast habits, and bread type) were collected from standardised questionnaires and clinical records and incorporated into downstream analyses (see Supplementary Methods, Table S2). The sample size was determined by the number of eligible participants available during the study period rather than by an a priori power calculation. The primary analysis was the comparison between HFpEF patients and healthy controls, whereas stratified analyses were considered secondary, exploratory, and hypothesis-generating. Healthy controls were recruited with the aim of achieving a comparable distribution of age and sex and thereby minimising potential confounding. Given the limited size of the stratified subgroups, the findings were interpreted cautiously, with effect sizes reported where appropriate to complement significance testing.

### 2.2. DNA Isolation and 16S rRNA Gene Sequencing

Genomic DNA was extracted using the GeneMATRIX Stool DNA Purification Kit (EURx, Gdansk, Poland). Samples were assessed for quality, quantified, and sent to Novogene for paired-end 16S ribosomal RNA (16S rRNA) gene sequencing (Illumina PE250) targeting the V3–V4 region, with at least 100,000 reads per sample. Because the obtained read lengths were insufficient to ensure reliable overlap of the paired-end reads, the downstream sequence-processing workflow was adapted to single-end analysis.

### 2.3. Sequence Processing, Diversity Analysis, and Taxonomy

Raw reads were processed in QIIME 2 (2025.7). Read quality was first assessed on a per-base basis using the demux summarise workflow. Denoising, error correction, and chimaera removal were performed using DADA2 (Divisive Amplicon Denoising Algordithm 2, denoise-single) to generate amplicon sequence variants. The first 10 bp was removed from the 5′ end (trim-left = 10), and reads were truncated at 400 bp (trunc-len = 400) based on the observed quality profiles. Three technical replicates per participant were assessed for consistency and subsequently merged into a single profile to increase sequencing depth and reduce variability. Alpha diversity was assessed using Observed Features (OF), Shannon diversity, Pielou’s evenness, and Faith’s phylogenetic diversity (Faith’s PD); beta diversity was assessed using Bray–Curtis, Jaccard, and UniFrac metrics (weighted—WU and unweighted—UU), with community differences tested using ADONIS. Taxonomic assignment (phylum to genus) was performed using the SILVA 138 V3–V4 classifier; mitochondrial, chloroplast, eukaryotic, and unassigned features were removed. Differential abundance was tested with ANCOM (Analysis of Composition of Microbiomes), applying prevalence filtering and biological plausibility checks for NYHA-stratified analyses. Candidate taxa were additionally evaluated for abundance distribution and consistency across groups to minimise spurious associations driven by rare taxa or outlier-dominated signals.

### 2.4. Functional Prediction (PICRUSt2)

Functional potential was inferred using PICRUSt2 via the Galaxy platform [10], generating unstratified Enzyme Commission (EC) and KEGG Ortholog (KO) tables. These outputs represent predicted functional profiles rather than direct metagenomic or experimental measurements of microbial functions and were therefore considered hypothesis-generating. PICRUSt2 outputs were post-processed to calculate group means, log_2_ fold changes, Welch’s *t*-test *p*-values, and false discovery rate (FDR)-adjusted q-values, retaining functions present in a minimum number of samples per group. Outliers were flagged using the MAX/LARGE ratio. Top differences were visualised as log_2_ fold-change bar plots. KO results are shown in the main figures, with raw KO data provided in the Supplementary Information, together with EC tables (Supplementary Tables S3–S8). Features were ranked by absolute log_2_ fold change, while statistical significance was evaluated using Benjamini–Hochberg FDR correction.

### 2.5. Statistical Analysis

Baseline characteristics were compared using independent-samples *t*-tests or chi-square tests (two-tailed, *p* < 0.05) in SPSS 20.0. GM analyses in QIIME2 used Kruskal–Wallis tests for alpha-diversity, ADONIS (PERMANOVA) for beta-diversity, and ANCOM for differential abundance. For alpha-diversity analyses involving multiple group comparisons, adjusted q-values were considered alongside nominal *p*-values. For the primary HFpEF-versus-control alpha-diversity comparisons, effect magnitude was additionally summarised using Kruskal–Wallis eta-squared (η^2^H). PICRUSt2 functional comparisons applied two-tailed *t*-tests with Benjamini–Hochberg FDR correction (q < 0.05). Welch’s *t*-test was used where unequal variances were present. ADONIS analyses were performed using 999 permutations.

Effect sizes for baseline clinical and echocardiographic characteristics were reported as Hedges’ g for continuous normally distributed variables, Cramér’s V for categorical variables, and rank-biserial correlation for continuous non-normally distributed variables. Positive Hedges’ g values indicate higher values in the HFpEF group, whereas negative values indicate higher values in healthy controls. For beta-diversity analyses, the ADONIS R^2^ statistic was used as the measure of effect magnitude, representing the proportion of community-composition variation explained by the tested variable or model. For ANCOM analyses, the W statistic and the corresponding decision regarding rejection of the null hypothesis were reported, together with percentile ranges of the abundance distributions for the reported taxa, as ANCOM does not provide a conventional standardised effect-size measure for compositional data. For PICRUSt2 analyses, log_2_ fold-change (log_2_FC) was used to describe the magnitude and direction of predicted functional differences. Secondary stratified analyses were considered exploratory and hypothesis-generating; therefore, their findings were interpreted cautiously, with effect sizes reported where appropriate to complement significance testing.

## 3. Results

### 3.1. Study Population: HFpEF vs. Healthy Controls

The HFpEF cohort comprised older adults and was predominantly female, with similar female representation in the HFpEF and control groups (~60%). Mean age was 74 ± 6 years in both groups, and controls were matched for age and sex, with negligible effect sizes for both variables (Supplementary Table S2). Patients with HFpEF had a higher BMI than healthy controls (29.6 ± 5.8 vs. 26.2 ± 3.6 kg/m^2^, *p* = 0.010; Hedges’ g = 0.689), while hypertension and diabetes mellitus (DM) showed moderate categorical effect sizes (Cramér’s V = 0.314 and 0.303, respectively). As expected, the largest between-group effects were observed for HF-related clinical and echocardiographic characteristics, including pulmonary artery systolic pressure (Hedges’ g = 1.605), left atrial volume index (Hedges’ g = 1.279), and posterior wall thickness (Hedges’ g = 1.235). Reduced estimated glomerular filtration rate and 6 min walking distance were also observed in HFpEF patients, with moderate and large effect sizes, respectively (Hedges’ g = −0.647 and −0.943). Complete effect-size estimates for the baseline clinical and echocardiographic characteristics are provided in Supplementary Table S2.

### 3.2. Gut Microbiota in the HFpEF Cohort

To evaluate whether GM composition differs between HFpEF and controls, faecal DNA from 30 HFpEF patients and 28 controls was subjected to 16S rRNA gene sequencing. Approximately 38,000,000 reads were generated, with consistently high sequencing depth (median ~160,000 reads/sample; no samples < ~100,000). Feature filtering removed predominantly low-abundance signals while retaining ~37,000,000 reads and stable median read counts, with no samples < ~90,000. Features with very low abundance (<10 reads) detected in only a single sample were excluded as being more consistent with sequencing artefacts than biological variation.

Technical replicates showed clustering by individual, confirming reproducibility. One control participant was excluded due to pronounced replicate divergence, while all remaining subjects demonstrated adequate concordance (Supplementary Figure S1). The final dataset comprised 30 HFpEF patients and 28 controls and was used for alpha- and beta-diversity analyses, taxonomic assignment, differential abundance testing (ANCOM), and functional inference (PICRUSt2).

#### 3.2.1. Gut Microbiota Diversity Landscape and Ecological Constraints in HFpEF

**Figure 1:**
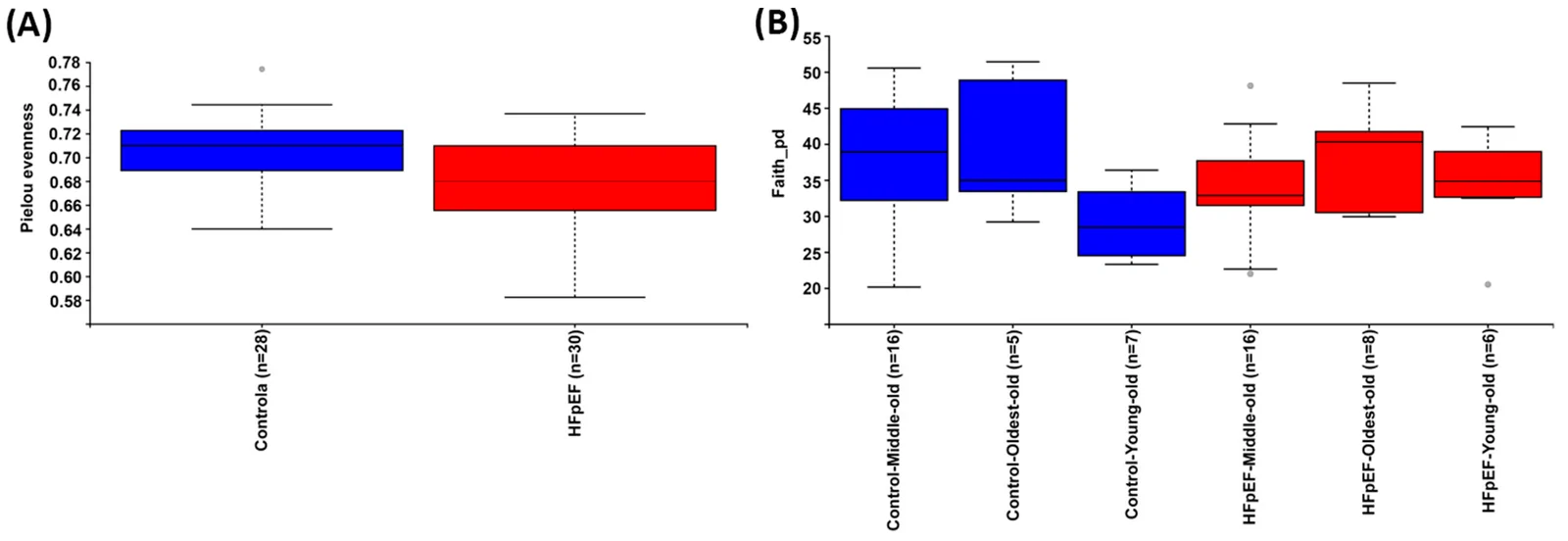
Alpha-diversity of gut microbiota (GM). (**A**) Pielou’s evenness, reflecting the distributional evenness of bacterial taxa within each sample, in controls (n = 28) and HFpEF patients (n = 30) (H = 4.22, *p* = 0.04, q = 0.04, η^2^H = 0.058); (**B**) Faith’s PD, a measure of within-sample phylogenetic diversity, across age groups in controls and HFpEF patients (n indicated per group). Boxplots show median, interquartile range, and range; dots indicate outliers.

Multiple alpha-diversity metrics were assessed to capture richness, evenness, and composite diversity. HFpEF patients consistently showed a tendency toward lower diversity compared with controls, although effects were metric-dependent and modest. Richness-based metrics did not differ significantly; however, Observed Features and Faith’s PD suggested a narrower range of taxonomic and phylogenetic richness in HFpEF. In contrast, Pielou’s evenness was significantly reduced in HFpEF patients compared with controls (Kruskal–Wallis H = 4.22, *p* = 0.04, q = 0.04, η^2^H = 0.058, Figure 1A), with lower central values and greater dispersion in HFpEF, indicating increased dominance of fewer taxa rather than a uniform loss of richness. Shannon diversity showed a similar trend (H = 3.04, *p* = 0.081, q = 0.081, η^2^H = 0.036).

Age and sex showed limited associations with alpha-diversity. Richness-based metrics showed a trend toward lower richness in the oldest participants, while evenness remained largely stable across categories. Shannon diversity showed only trend-level effects in disease-stratified middle-aged groups, and no significant associations were observed with sex (Figure 1B). Pairwise contrasts indicated greater richness variability among the oldest participants, although global effects were not significant.

**Figure 2:**
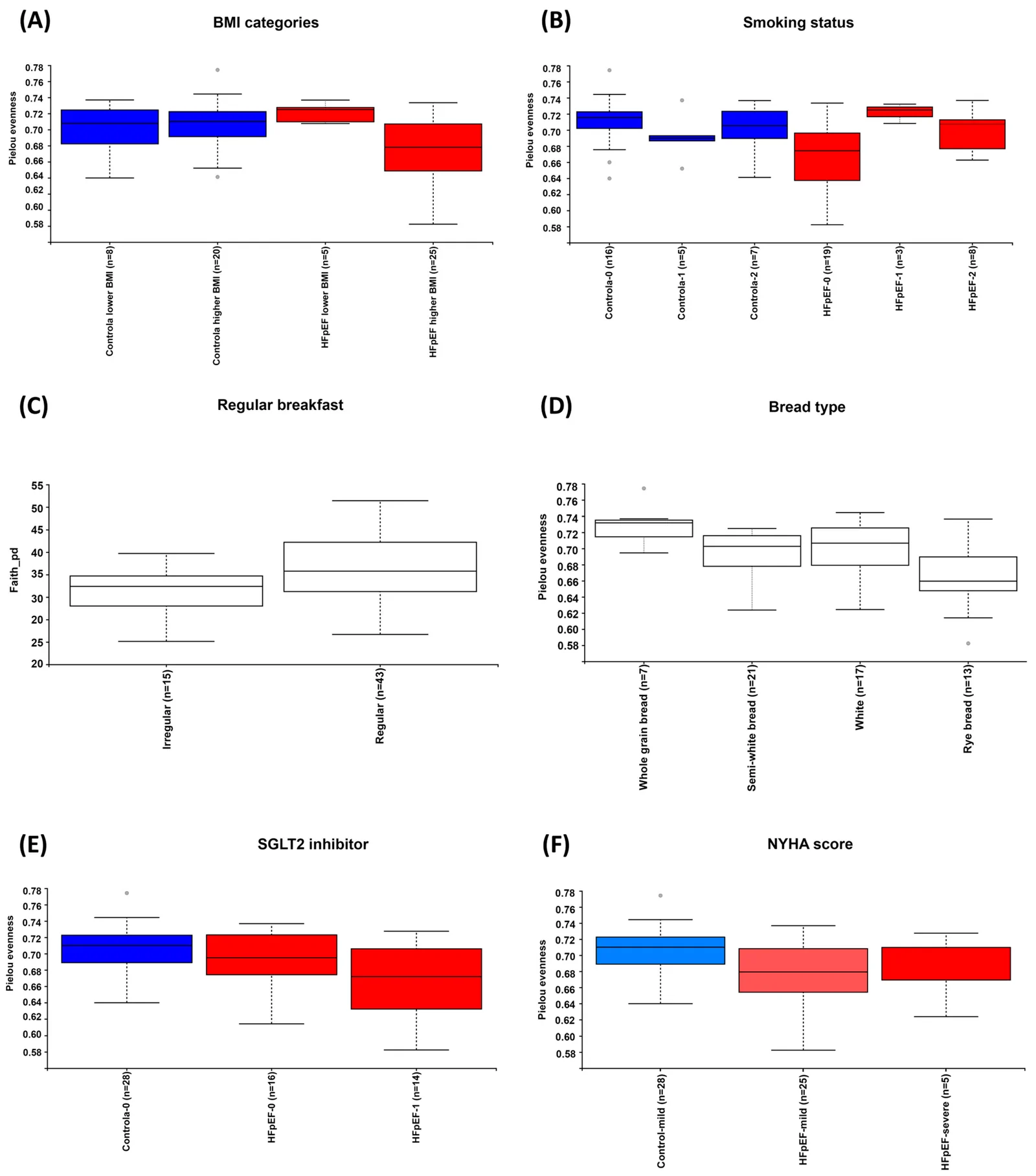
Alpha-diversity of gut microbiota (GM) across metabolic, lifestyle, and clinical factors. (**A**) Pielou’s evenness across BMI categories (H = 12.42, *p* = 0.006); (**B**) Pielou’s evenness by smoking status (H = 12.7, *p* = 0.03); (**C**) observed features (OF) by breakfast regularity (H = 5.59, *p* = 0.02) and Faith’s PD (H = 4.66, *p* = 0.03); (**D**) Pielou’s evenness by bread type (H = 12.61, *p* = 0.006); (**E**) Pielou’s evenness by SGLT2 inhibitor use (H = 7.64, *p* = 0.02); (**F**) Pielou’s evenness across NYHA functional class (trend-level differences, non-significant after multiple testing correction). n indicated per group. Boxplots show median, interquartile range, and range; dots indicate outliers.

In contrast, metabolic and lifestyle factors showed stronger associations with alpha-diversity than age or sex. BMI was unrelated to richness, but evenness differed significantly across BMI–disease strata (H = 12.42, *p* = 0.006, Figure 2A), driven primarily by higher-BMI HFpEF patients, who showed lower central evenness and greater dispersion; Shannon diversity showed a similar pattern (H = 9.44, *p* = 0.02). BSA showed no consistent associations, whereas smoking status was associated with evenness differences (H = 12.7, *p* = 0.03, Figure 2B), mainly between non-smoking controls and HFpEF patients, while richness and Shannon diversity remained unchanged.

Dietary habits were analysed as coarse indicators of habitual patterns. Regular breakfast consumption was associated with richness (OF: H = 5.59, *p* = 0.02; Faith’s PD: H = 4.66, *p* = 0.03, Figure 2C), with irregular breakfast intake linked to lower richness and reduced dispersion, whereas evenness and Shannon diversity were unaffected. Bread type showed the strongest dietary association, with significant differences in evenness (H = 12.61, *p* = 0.006, Figure 2D) and Shannon diversity, including multiple pairwise contrasts surviving multiple-testing correction, while richness showed only trend-level pairwise differences. Fat type and salt addition were not associated with overall alpha-diversity, apart from isolated trend-level pairwise effects.

To distinguish GM variation related to host metabolic dysregulation from external lifestyle factors, alpha-diversity was further examined according to DM, hyperlipidaemia (HLP), and sodium–glucose cotransporter 2 (SGLT2) inhibitor use within control and HFpEF strata. Neither DM nor HLP showed consistent associations with alpha-diversity, except for a trend-level evenness difference between controls without and HFpEF patients with DM. In contrast, SGLT2 inhibitor use was associated with evenness (H = 7.64, *p* = 0.02, Figure 2E), whereas richness remained unchanged; significant pairwise difference after multiple-testing correction was observed between untreated controls and treated HFpEF patients, with the latter showing a greater dispersion of evenness values.

NYHA functional class was evaluated as a downstream marker of disease expression rather than an etiological driver. Richness metrics showed no differences, and Shannon diversity remained unchanged. A trend-level pairwise difference in evenness was observed between controls and HFpEF patients with mild NYHA class, although it did not survive multiple-testing correction; mild HFpEF was characterised by lower central evenness and greater dispersion (Figure 2F).

Across beta-diversity metrics, no clear clustering of HFpEF patients and controls was observed, consistent with ADONIS analysis showing no detectable effect of HFpEF status on global community structure. Age showed the largest, although modest, contribution among the tested covariates, particularly across UU and Bray–Curtis distances, in line with the alpha-diversity findings. Other individual covariates and selected combinations of covariates explained only a small proportion of the variation (R^2^ < 6%), with approximately 90–97% of the variation remaining unexplained across the different beta-diversity metrics and model specifications.

#### 3.2.2. Gut Microbiota Taxonomic Composition in HFpEF

**Figure 3:**
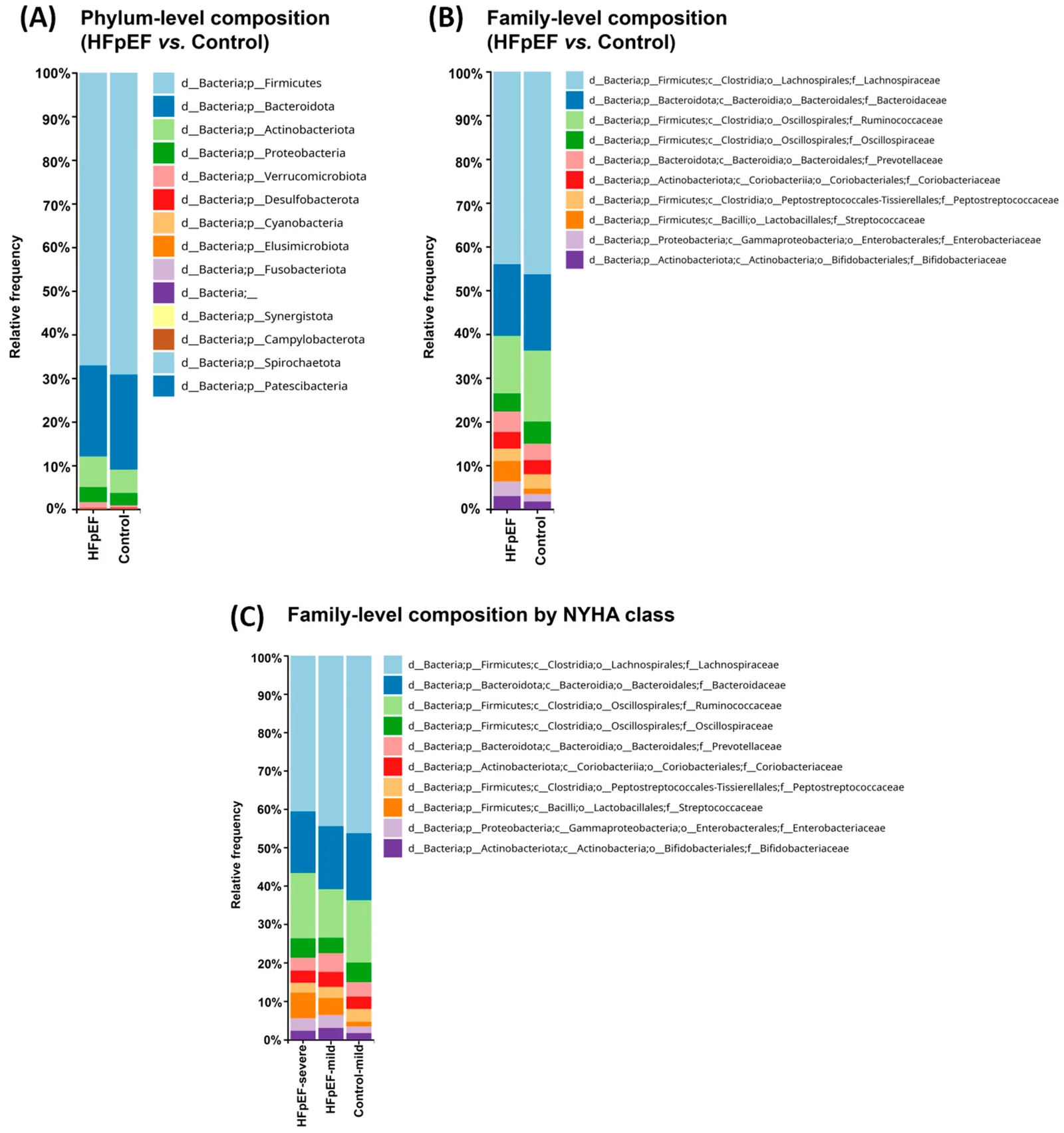
Relative abundance of bacterial taxa in patients with HFpEF compared with the control group. (**A**) Gut microbiota (GM) composition at the phylum level in HFpEF and control groups; (**B**) GM composition at the family level in HFpEF and control groups; (**C**) relative abundance of dominant bacterial taxa at the family level stratified by NYHA functional class (HFpEF severe, HFpEF mild, and control). The figure displays the top 10 most abundant taxa, shown as relative abundances (%).

At phylum level (L2), ~90% of sequences in both groups were assigned to *Firmicutes* and *Bacteroidota*, with a slightly lower proportion in HFpEF and a slightly higher proportion in controls (Figure 3A). At family level, *Lachnospiraceae* was most abundant in both groups, modestly higher in controls, followed by *Bacteroidaceae* with comparable proportions. *Ruminococcaceae* and *Oscillospiraceae* were also dominant and visually more abundant in controls. *Prevotellaceae* and *Coriobacteriaceae* appeared relatively more abundant in HFpEF. Other abundant families, including *Streptococcaceae*, *Enterobacteriaceae*, and *Bifidobacteriaceae*, were descriptively enriched in HFpEF based on relative abundance profiles (Figure 3B).

Differential abundance testing identified no taxa differing between HFpEF and controls at the predefined thresholds, and demographic or clinical stratifications likewise yielded no robust differences. NYHA functional class was the only variable associated with taxonomic shifts, prompting focused analyses across controls, HFpEF-mild, and HFpEF-severe groups (Figure 3C). At the family level, several ANCOM-identified taxa showed progressive, although not uniformly linear, abundance shifts with increasing functional limitation (Table 1).

*Lachnospiraceae* and *Ruminococcaceae* showed stepwise decreases from controls to mild HFpEF and further to severe HFpEF, indicating progressive depletion with advancing functional impairment (*Lachnospiraceae*: 1 → 0.96 → 0.85; *Ruminococcaceae*: 1 → 0.76 → 0.60). A similar progressive decline was observed for *Butyricicoccaceae* (1 → 0.60 → 0.30). *Bacteroidaceae* remained stable in mild HFpEF but declined markedly in severe HFpEF (1 → 1 → 0.45). In contrast, *Akkermansiaceae* increased strongly across strata (1 → 4.3 → 17.3), with the highest median abundance in severe HFpEF. *Enterobacteriaceae* showed a non-linear pattern, expanding in NYHA-mild HFpEF (1 → 6.8) and decreasing in NYHA-severe HFpEF (0.42), consistent with a transient expansion rather than sustained enrichment. Additional ANCOM-identified families showed similar but more moderate or less consistent NYHA-associated shifts (Supplementary Table S9).

#### 3.2.3. Predicted Microbial Functional Profiles (PICRUSt2)

**Figure 4:**
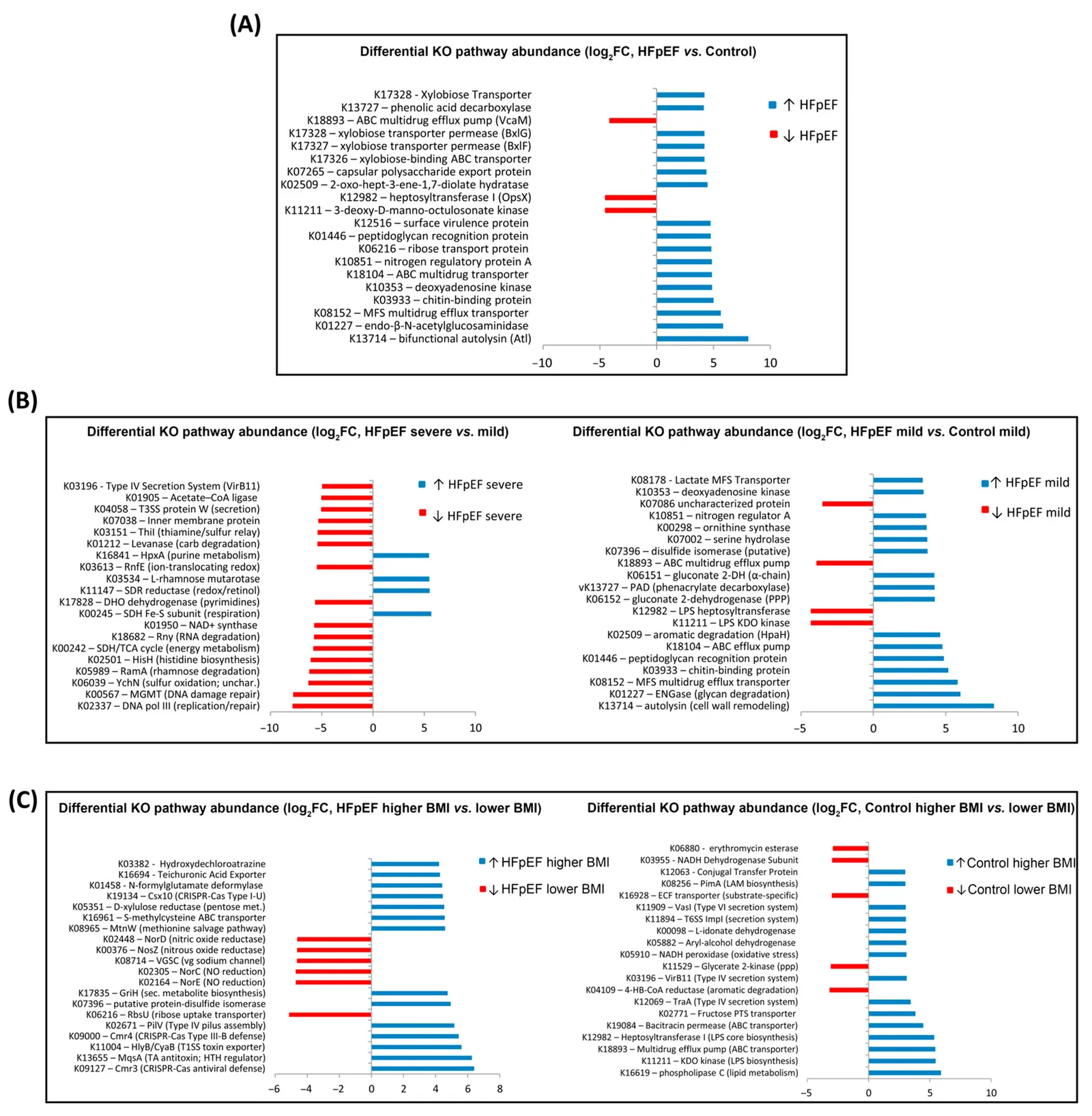
Predicted functional profiles of GM based on PICRUSt2 analysis. The 20 functions with the largest absolute log_2_ fold changes are shown for each comparison. (**A**) HFpEF vs. controls; (**B**) NYHA-severe vs. NYHA-mild HFpEF and NYHA-mild HFpEF vs. controls; (**C**) higher- vs. lower-BMI HFpEF and higher- vs. lower-BMI controls. Positive log_2_FC values indicate higher predicted abundance in the first group named in each comparison, whereas negative values indicate lower predicted abundance.

PICRUSt2 predicted >6000 KOs and ~1800 EC functions across samples. Because PICRUSt2 infers functional potential from 16S rRNA gene profiles, these results are interpreted as hypothesis-generating rather than direct measures of microbial function. After prevalence filtering and removal of extreme outliers, differential analyses were performed for HFpEF vs. controls, NYHA-severe vs. NYHA-mild HFpEF, NYHA-mild HFpEF vs. controls, and higher- vs. lower-BMI groups within HFpEF and controls. For each comparison, the 20 functions with the largest absolute log_2_ fold changes were visualised (Figure 4), with positive and negative log_2_FC values indicating enrichment and depletion, respectively, in the first group named in each comparison.

##### HFpEF vs. Controls

In the primary HFpEF-versus-control comparison, HFpEF exhibited a predicted functional profile consistent with stress adaptation and competitive fitness, rather than broad inflammatory activation (Figure 4A). Enriched functions included cell wall turnover, Major Facilitator Superfamily and ATP-binding cassette transporter systems, alternative carbohydrate utilisation, and aromatic compound degradation. Several pathways involved in lipopolysaccharide (LPS) biosynthesis were reduced, arguing against an endotoxin-dominant signature. Controls showed a more metabolically balanced profile with limited enrichment of stress pathways.

##### HFpEF: Severe vs. Mild

Within the HFpEF cohort, NYHA-severe HFpEF exhibited depletion of core metabolic pathways (energy, nucleotide, vitamin/cofactor metabolism) and reduced fermentative capacity, along with enrichment of oxidative stress responses and alternative metabolic routes (Figure 4B). This pattern is consistent with metabolic constraint and adaptation to an inflammatory or hypoxic environment. NYHA-mild HFpEF maintained a comparatively preserved and metabolically active profile.

##### NYHA-Mild HFpEF vs. Controls

In the comparison between NYHA-mild HFpEF and controls, NYHA-mild HFpEF showed moderate functional shifts compared to controls, including enrichment of glycan and aromatic compound degradation, efflux-associated stress responses, and pentose phosphate activity (Figure 4B). LPS biosynthesis pathways remained reduced, suggesting that endotoxin may play a limited role in early disease changes.

##### HFpEF: Higher BMI vs. Lower BMI

Within HFpEF, higher BMI exhibited a pronounced stress-adapted profile (Figure 4C), with enrichment of CRISPR–Cas, toxin–antitoxin systems, and secretion/export pathways, as well as altered sulphur/methionine redox metabolism and reduced nitric oxide reduction. Lower BMI showed relatively preserved metabolic potential.

##### Controls: Higher BMI vs. Lower BMI

In controls, higher BMI was associated with enrichment of LPS biosynthesis, membrane remodelling, secretion systems, and nutrient transport, consistent with low-grade inflammatory and competitive traits. Lower BMI controls retained a more redox-balanced profile with preserved denitrification pathways.

## 4. Discussion

HFpEF remains a heterogeneous syndrome with limited disease-modifying therapies, highlighting the need for pathophysiology-driven targets. Chronic low-grade inflammation and cardiometabolic stress place the GM at the interface of metabolic, immune, and cardiovascular pathways. Our data indicate that HFpEF is not characterised by a uniform dysbiotic signature, but rather by context-dependent microbial reorganisation linked to metabolic burden and disease severity.

The clinical characteristics of the HFpEF cohort, including older age, a female predominance, frequent overweight, hypertension, and DM, were consistent with the established HFpEF phenotype [11]. Echocardiographic findings and medical therapy likewise reflected current diagnostic criteria and guideline-based management [12,13,14]. Although BMI, diabetes, and HFpEF-related medication use differed between groups, these factors are closely linked to the clinical characteristics of HFpEF and are difficult to fully disentangle in a small cohort. This is particularly relevant in older adults, in whom age-related changes in gut microbiota may interact with multimorbidity, diet, medication use, physical activity, and other factors associated with healthy or unhealthy ageing [15,16,17,18,19]. Major additional sources of microbiota-related confounding were minimised through the study exclusion criteria, while exploratory analyses of demographic, clinical, treatment-related, and dietary variables showed only modest contributions to global community structure. Similarly, factors contributing to obesity-related heterogeneity, including comorbidities, fatigue, and depressive symptoms, were not systematically assessed and may represent additional sources of residual confounding [20]. Depression in older adults may also be associated with less favourable health behaviours, increased cardiovascular risk, and inflammatory alterations, further underscoring the clinical complexity of this population [17,21]. Nevertheless, residual confounding could not be excluded given the limited sample size.

At the cohort level, HFpEF was associated with modest changes in alpha-diversity, primarily affecting evenness, whereas global beta-diversity remained unchanged. Age showed the largest, although modest, contribution among the tested covariates to community structure, whereas most of the observed variation remained unexplained by the variables included in the exploratory models. The greater variability observed among older participants is consistent with previous reports linking ageing to reduced gut microbial ecological stability under chronic physiological stress [15]. Sex-related signals were minimal, likely reflecting postmenopausal status [22].

Metabolic burden emerged as a key modifier of GM organisation. BMI correlated with evenness and Shannon diversity, indicating that higher BMI status reshapes relative abundance rather than richness, producing heterogeneous but potentially stable configurations under metabolic stress [23]. BSA showed no consistent effect, supporting adiposity-related metabolic pressure as the main driver. Smoking influenced community balance and may partially mask disease-associated signals, consistent with evidence that tobacco exposure alters GM-composition without uniform taxon loss [24].

Dietary exposures represented an additional ecological pressure on the GM. Regular breakfast consumption was associated primarily with richness-based metrics, without consistent changes in evenness, in line with effects linked to short-term feeding patterns and circadian influences rather than long-term restructuring of the microbial community [25]. In contrast, bread type showed stronger associations with evenness and Shannon diversity, with minimal differences in richness, indicating potential shifts in relative abundance structure and competitive dynamics within an otherwise stable taxonomic pool. As a proxy for fermentable carbohydrates and dietary fibre availability, bread may reflect substrate-driven ecological constraints shaping GM-organisation [26,27]. Although dietary resolution was limited, the consistency of obtained associations supports dietary composition as a relevant axis for GM-modulation. Fat type and salt showed minimal influence, consistent with limited fermentable substrates and coarse exposure measures [28,29]. Overall, dietary variables reflecting carbohydrate and fibre intake showed the strongest associations with GM organisation.

Among clinical factors, DM and HLP minimally influenced alpha diversity, whereas SGLT2 inhibitor use selectively affected evenness, potentially reflecting indirect ecological effects via systemic metabolic and inflammatory modulation [30].

ANCOM did not identify significantly differentially abundant taxa between HFpEF patients and healthy controls, despite the observed differences in community evenness and the trends in overall diversity. This absence of a uniform taxonomic signature may partly reflect substantial inter-individual variability in GM composition, especially among older adults [15,16], together with the limited sample size and the presence of comorbidities, medications, and other lifestyle-related factors that are common in an older HFpEF population. Thus, diagnosis alone may not capture the ecological heterogeneity of the GM in HFpEF.

NYHA functional class was the only stratification yielding reproducible differential abundance signals, suggesting that GM changes in HFpEF track disease severity more closely than diagnosis alone. Severity-associated patterns included depletion of *Lachnospiraceae*, *Ruminococcaceae*, and *Butyricicoccaceae*, enrichment of *Akkermansiaceae*, and a non-linear *Enterobacteriaceae* expansion in milder HFpEF followed by reduction in severe disease. The depletion of these SCFA-associated and fermentative taxa aligns with previous HFpEF studies [9,31] and parallels the predicted reduction in SCFA fermentation pathways in NYHA-severe HFpEF. This convergence of taxonomic and functional predictions may indicate a progressive reduction in microbial metabolic capacity with increasing functional limitation, although the predicted nature of the functional analysis precludes direct inference of SCFA production. *Bacteroidaceae* decline may further reflect reduced fermentative capacity [32]. *Enterobacteriaceae* dynamics may reflect a context-dependent expansion of opportunistic taxa under altered metabolic conditions rather than fixed pathobiont overgrowth. Experimental evidence indicates that changes in the intestinal metabolic environment, including increased availability of substrates such as TMAO, can preferentially stimulate the growth and metabolic activity of *Enterobacteriaceae* [33]. In the present cross-sectional cohort, however, this non-linear pattern cannot establish whether the observed expansion in milder HFpEF represents a transient response to metabolic stress or a disease-stage-specific association.

PICRUSt2 functional inference supported the taxonomic findings. Together with the limited taxonomic differences, these findings suggest that functional adaptation may be more prominent than broad compositional restructuring in HFpEF. Compared with controls, HFpEF showed predicted enrichment of pathways related to cell wall turnover, multidrug transport, and alternative carbohydrate utilisation, reflecting stress adaptation rather than broad inflammatory activation [31]. Multiple lipopolysaccharide biosynthesis signals were reduced, suggesting that endotoxin may play a limited role. NYHA-severe HFpEF exhibited depletion of pathways related to core metabolic processes and SCFA fermentation, consistent with metabolic exhaustion and with reduced epithelial and immune homeostasis [34]. Higher BMI status amplified predicted stress-adapted signatures (CRISPR-Cas, toxin–antitoxin regulation, efflux/secretion systems) and reduced nitric oxide detoxification, while lower BMI HFpEF patients retained relatively preserved metabolic potential; obesity in controls showed similar low-grade inflammatory traits [35]. A modest increase in trimethylamine-related pathways in NYHA-severe HFpEF aligns with links to systemic trimethylamine N-oxide and microvascular dysfunction [36], requiring further validation.

Clinically, these results support a severity-informed perspective: HFpEF does not appear to be associated with a uniform dysbiotic state in older adults, but interacts with the GM along gradients of metabolic burden and functional limitation. These findings support exploration of strategies aimed at the microbial metabolic environment, including dietary interventions and prebiotics, rather than approaches focused solely on normalising community composition. However, no interventional data on diet, probiotics, or prebiotics were collected in the present cohort, and these therapeutic approaches therefore remain speculative. This study is limited by its moderate sample size, cross-sectional design precluding causal inference, and reliance on predicted rather than directly measured microbial function. Larger longitudinal and interventional studies with metabolomic validation are needed to clarify causality, identify intervention-responsive features, and determine which HFpEF phenotypes may derive the greatest benefit from microbiota-targeted interventions.

## Figures and Tables

**Table 1:** Median feature abundances of ANCOM-flagged bacterial families across NYHA functional classes. Selected families identified as differentially abundant by ANCOM are shown across controls, HFpEF-mild (NYHA I–II), and HFpEF-severe (NYHA III–IV) groups. Fold change values indicate relative differences in median abundance across strata.

Family	Median (Control)	Median (HFpEF-Mild)	Median (HFpEF-Severe)	Fold Change (Control → Mild → Severe)
*Lachnospiraceae*	188,186	180,023	159,867	1 → 0.96 → 0.85
*Ruminococcaceae*	72,830	55,014	43,848	1 → 0.76 → 0.6
*Enterobacteriaceae*	362	2468	153	1 → 6.8 → 0.42
*Bacteroidaceae*	54,160	54,492	24,231	1 → 1 → 0.45
*Akkermansiaceae*	79	344	1366	1 → 4.3 → 17.3
*Butyricicoccaceae*	3640	2193	1084	1 → 0.6 → 0.3

## Data Availability

Data available upon request. The SILVA reference database/classifier used for taxonomic classification is publicly available through the SILVA database.

## Supplementary Materials

The following supporting information can be downloaded at https://www.mdpi.com/article/10.3390/biomedicines14092123/s1, Supplementary Methods; Figure S1: Technical replicate concordance (Emperor plot); Table S1: Inclusion and exclusion criteria (in Supplementary Methods); Table S2: Characteristics of HFpEF patients and healthy controls (in Supplementary Methods); Table S3: PICRUSt2 EC functions—HFpEF vs. Controls; Table S4: PICRUSt2 EC functions—HFpEF severity (NYHA); Table S5: PICRUSt2 EC functions—BMI categories; Table S6: PICRUSt2 KO functions—HFpEF vs. Controls; Table S7: PICRUSt2 KO functions—HFpEF severity (NYHA); Table S8: PICRUSt2 KO functions—BMI categories; Table S9: ANCOM differentially abundant bacterial families across NYHA functional classes.

## Abbreviations

The following abbreviations are used in this manuscript:

HF	Heart failure
HFrEF	Heart failure with reduced ejection fraction
HFmrEF	Heart failure with mildly reduced ejection fraction
HFpEF	Heart failure with preserved ejection fraction
LVEF	Left ventricular ejection fraction
GM	Gut microbiota
DM	Diabetes mellitus
HLP	Hyperlipidaemia
BMI	Body mass index
BSA	Body surface area
NYHA	New York Heart Association
SGLT2	Sodium–glucose cotransporter 2
16S rRNA	16S ribosomal ribonucleic acid
DNA	Deoxyribonucleic acid
QIIME2	Quantitative insights into microbial ecology 2
DADA2	Divisive amplicon denoising algorithm 2
ASV	Amplicon sequence variant
OF	Observed features
PD	Phylogenetic diversity
UU	Unweighted UniFRac
WU	Weighted UniFRac
ANCOM	Analysis of composition of microbiomes
PICRUSt2	Phylogenetic investigation of communities by reconstruction of unobserved states 2
KO	KEGG ortholog
EC	Enzyme commission
FDR	False discovery rate
SCFA	Short-chain fatty acids
LPS	Lipopolysaccharide
