# Supplementary material for: Gut Microbiota in Heart Failure with Preserved Ejection Fraction: Clinical Phenotyping and Therapeutic Opportunities
Source: Biomedicines. 2026 Sep 19;14(9):2123. doi: 10.3390/biomedicines14092123 (PMC13604396; doi:10.3390/biomedicines14092123)
Supplement: Supplementary file 1 [file biomedicines-14-02123-s001.zip › biomedicines-4556612 Supplementary_Methods.pdf]

**Supplementary Methods**

**S1. Study population and sample collection**

This study analysed faecal DNA from a clinical cohort of 60 individuals, comprising 30 HfpEF patients HFpEF and 30 healthy controls. Study inclusion/exclusion criteria are listed in Table S1. Two control participants were excluded; one due to technical issues with DNA extraction and the other due to divergent sequencing replicates, resulting in 58 subjects. Detailed clinical metadata were collected: demographic, anthropometric, clinical, echocardiographic, laboratory, and functional characteristics. For subgroup analyses, participants were stratified by BMI (BMI $<25$ kg/$m^2$ classified as lean, and $\geq 25$ kg/$m^2$ as obese). NYHA functional classes I–II were grouped as mild, while classes III–IV as severe due to the sample size distribution.

Demographic, metabolic, and lifestyle data – including BMI, BSA, smoking status, SGLT2 inhibitor use, breakfast habits, and bread type – were collected using standardised questionnaires and clinical records and included in downstream analyses (Table S2).

**Table S1**. Inclusion and exclusion criteria.

| Inclusion criteria for the study cohort | Exclusion criteria |
|---|---|
| - Written informed consent obtained;<br>- Symptoms of heart failure;<br>- Left ventricular ejection fraction (LVEF) ≥ 50% and:<br>NT-proBNP > 125 pg/mL or BNP > 35 pg/mL in sinus rhythm,<br>or,<br>in the presence of atrial fibrillation, NT-proBNP > 365 pg/mL or BNP > 105 pg/mL.<br>- At least one of the following criteria:<br>- Left atrial volume index (LAVI) > 34 mL/$m^2$ in sinus rhythm or > 40 mL/$m^2$ in atrial fibrillation (or alternatively, left atrial diameter > 40 mm),<br>- Left ventricular mass index ≥ 95 g/$m^2$ in women or ≥ 115 g/$m^2$ in men,<br>- Septal e′ < 7 cm/s,<br>- Lateral e′ < 10 cm/s,<br>- E/e′ ratio > 15. | - Active malignancy;<br>- Chronic gastrointestinal diseases;<br>- Antibiotic or probiotic therapy within the previous three months;<br>- Abdominal surgery within the previous three months;<br>- Restrictive cardiomyopathy or arrhythmogenic right ventricular cardiomyopathy; cardiac amyloidosis, sarcoidosis, endomyocardial fibrosis;<br>- Hypertrophic cardiomyopathy;<br>- Constrictive pericarditis or active peri/myocarditis;<br>- Left ventricular ejection fraction (LVEF) < 50%<br>- Autoimmune diseases and systemic connective tissue disorders;<br>Severe chronic kidney disease, eGFR < 30 mL/min. |

**Table S2**. Characteristics of HFpEF patients and healthy controls.

| Variable | HFpEF (N=30) | Healthy controls (N=28) | p |
|---|---|---|---|
| Age, mean ± SD (range) | 74 ± 6 (61–83) | 74 ± 6 (62–84) | 0.362 |
| Females, N (%) | 18 (60.0) | 17 (60.7) | 0.096 |
| BMI (kg/m²) | 29.6 ± 5.8 | 26.2 ± 3.6 | 0.010 |
| BSA (m²) | 1.96 ± 0.30 | 1.86 ± 0.21 | 0.138 |
| CAD, N (%) | 7 (23.3) | 2 (7.1) | 0.089 |
| PCI, N (%) | 2 (6.7) | 2 (7.1) | 0.943 |
| Hypertension, N (%) | 29 (96.7) | 21 (75.0) | 0.017 |
| Diabetes mellitus, N (%) | 11 (36.7) | 3 (10.7) | 0.021 |
| Dyslipidaemia, N (%) | 20 (66.7) | 19 (67.9) | 0.923 |
| Current smoker, N (%) | 3 (10.0) | 5 (17.9) | 0.685 |
| COPD, N (%) | 2 (4.7) | 1 (3.6) | 0.595 |
| SGLT2i, N (%) | 14 (46.7) | 0 (0) | <0.001 |
| Furosemid, N (%) | 14 (46.7) | 0 (0) | <0.001 |
| ACEi, N (%) | 15 (50) | 12 (42.9) | 0.586 |
| MRA, N (%) | 10 (33.3) | 0 (0) | 0.001 |
| Statins, N (%) | 17 (56.3) | 13 (46.4) | 0.46 |
| Systolic BP (mmHg) | 143 ± 24 | 139 ± 17 | 0.437 |
| Diastolic BP (mmHg) | 82 ± 13 | 80 ± 11 | 0.500 |
| LV EDD (mm) | 47 ± 6 | 44 ± 5 | 0.033 |
| LV ESD (mm) | 32 ± 7 | 30 ± 4 | 0.043 |
| LV EF (%) | 56 ± 5 | 58 ± 5 | 0.053 |
| IVSd (mm) | 11 ± 2 | 10 ± 1 | 0.005 |
| PWd (mm) | 11 ± 2 | 9 ± 1 | 0.008 |
| LVMi | 95 ± 29 | 76 ± 14 | 0.002 |
| E (m/s) | 0.85 ± 0.21 | 0.66 ± 0.18 | <0.001 |
| A (m/s) | 0.94 ± 0.15 | 0.78 ± 0.25 | 0.016 |
| E’ lateral (m/s) | 0.11 ± 0.04 | 0.12 ± 0.02 | 0.695 |
| E’ medial (m/s) | 0.10 ± 0.09 | 0.08 ± 0.02 | 0.190 |
| E/e’ | 9.0 ± 2.4 | 6.8 ± 1.9 | <0.001 |
| LAd (mm) | 40 ± 8 | 34 ± 4 | <0.001 |
| LAVi (ml/m²) | 41 ± 15 | 26 ± 6 | <0.001 |
| TAPSE (mm) | 23 ± 6 | 23 ± 4 | 0.628 |
| PASP (mmHg) | 32 ± 10 | 19 ± 5 | <0.001 |
| Haemoglobin (g/L) | 144 ± 13 | 143 ± 12 | 0.674 |
| CRP (mg/L) | 1.7 (0.75–5.4) | 1.9 (0.80–2.3) | 0.544 |
| NT-proBNP (pg/mL) | 370 (232–843) | 145 (90–165) | <0.001 |
| eGFR | 66.3 ± 17.5 | 77.1 ± 15.3 | 0.015 |
| Total cholesterol | 4.7 ± 1.2 | 5.6 ± 1.4 | 0.007 |
| 6MWT distance | 453 ± 80 | 525 ± 70 | 0.001 |
| Regular breakfast, N (%) | 20 (69.0) | 22 (78.6) | 0.506 |
| Dark bread, N (%) | 2 (6.9) | 5 (17.9) | 0.208 |

| | | | |
|---|---|---|---|
| Partially refined white bread, N (%) | 11 (37.9) | 10 (35.7) | 0.940 |
| Whole wheat bread, N (%) | 8 (27.6) | 9 (32.1) | 0.940 |
| Rye bread, N (%) | 8 (27.6) | 4 (14.3) | 0.245 |

Data are presented as mean ± SD, N (%) or median (IQR) as appropriate. **Abbreviations:** ACEi – angiotensin-converting enzyme inhibitors; BMI – body mass index; BSA – body surface area; CAD – coronary artery disease; COPD – chronic obstructive pulmonary disease; CRP – C-reactive protein; eGFR – estimated glomerular filtration rate; LV EDD – left ventricle end-diastolic diameter; LV ESD – left ventricle end-systolic diameter; IVSd – interventricular septum diameter; LAd – left atrium diameter; LAVi – left atrium volume index; LVMi – left ventricle mass index; MRA – mineralocorticoid receptor antagonist; PASP – pulmonic artery systolic pressure; PCI – percutaneous coronary intervention; PWd – posterior wall diameter; SGLT2i – sodium-glucose transporter 2 inhibitor; TAPSE – tricuspid annular plane systolic excursion.

## S2. DNA isolation and 16S rRNA gene sequencing

Genomic DNA was extracted using the GeneMATRIX Stool DNA Purification Kit (E3575-01; EURx, Poland). After quality assessment and quantification, samples were shipped to Novogene for paired-end 16S ribosomal sequencing (Illumina PE250), targeting the V3–V4 region, with a minimum depth of 100,000 reads per sample. Because read lengths were shorter than expected, downstream processing parameters were adjusted to ensure robust overlap and quality across the V3–V4 amplicon.

## S3. Sequence processing, diversity analysis, and taxonomy

Raw reads were processed in QIIME2 (qiime2-amplicon-2025.7) using DADA2 for error modelling, denoising, and chimera removal to generate amplicon sequence variants. Three technical replicates *per* participant were assessed for consistency using Emperor plots and merged into a single subject-level profile to increase depth and reduce intra-individual variability. Alpha-diversity was assessed using Observed Features (OF), Shannon diversity, Pielou's evenness, and Faith's phylogenetic diversity (Faith's PD). Beta-diversity was assessed using Bray–Curtis, Jaccard, and unweighted (UU) and weighted UniFrac (WU) metrics, while global community differences were tested using permutational multivariate analysis of variance (ADONIS). All analyses used a standardised sampling depth selected to optimise read retention without sample loss. Taxonomy (phylum to genus) was assigned using a pre-trained SILVA 138 (99%) V3–V4 classifier (SILVAdb v138.2). Mitochondrial,

chloroplast, eukaryotic, and unassigned features were removed. Differential abundance testing used ANCOM and was interpreted conservatively given compositionality and small subgroup sizes. For NYHA–stratified analyses, additional prevalence filtering and biological plausibility checks were applied to reduce spurious associations.

**S4. Functional prediction (PICRUSt2)**

Functional potential was inferred using PICRUSt2 *via* the publicly accessible Galaxy implementation (https://www.nature.com/articles/s41587-020-0548-6; Douglas et al., Nat Biotechnol 2020), generating unstratified Enzyme Commission (EC) and KEGG Ortholog (KO) tables. Outputs were exported and processed in Microsoft Excel (Microsoft Office 365). To reduce sparsity and low-abundance noise, functions were retained only if present in a minimum number of samples *per* group (≥10 of 30, ≥3 of 5, or ≥5 of 8, depending on group size). Potential outliers were flagged using the MAX/LARGE ratio. For each comparison, group means, $log_2$ fold changes, two-tailed Welch's t-test p-values, and Benjamini–Hochberg false discovery rate (FDR) corrections were computed. Top differences were visualised as $log_2$ fold-change bar plots. KO results are presented in the main figures and EC results in the SI (Supplementary Tables S3 – S5). Given the dimensionality and variability of PICRUSt2 predictions, most features did not remain significant after FDR correction; therefore, results were ranked primarily by absolute $log_2$ fold change (effect size), with full p and FDR values reported (Supplementary Tables S6 – S8). Findings were interpreted as broader functional trends rather than pathway-level measurements.

**S5. Statistical analysis**

Baseline characteristics were compared using independent-samples t-tests or chi-square tests, as appropriate (two-tailed; $p < 0.05$), using Statistical Package for Social Sciences (release 20.0, SPSS Inc, NY, USA). GM analyses were performed in QIIME2: alpha-diversity differences were tested using Kruskal–Wallis, beta-diversity using ADONIS, and differential abundance using ANCOM. PICRUSt2 functional comparisons used two-tailed t-tests with Benjamini–Hochberg FDR correction; statistical significance threshold was $q < 0.05$. For comparisons with unequal group sizes (e.g., severe *vs.* mild NYHA; obese *vs.* lean), Welch's two-sample t-test was used for not assuming equal variances or sample sizes.
