# Supplementary material for: Gut Microbiota in Heart Failure with Preserved Ejection Fraction: Clinical Phenotyping and Therapeutic Opportunities
Source: Biomedicines. 2026 Sep 19;14(9):2123. doi: 10.3390/biomedicines14092123 (PMC13604396; doi:10.3390/biomedicines14092123)

Supplementary Figure S1. Emperor plot showing clustering of technical replicates by individual. Assessment of technical replicate consistency across subjects. Technical replicates were clustered by individual, demonstrating high concordance and reproducibility. One control sample was excluded due to substantial divergence between its replicates (highlighted in green), while all other subjects displayed consistent replicate clustering, confirming the reliability of the data.

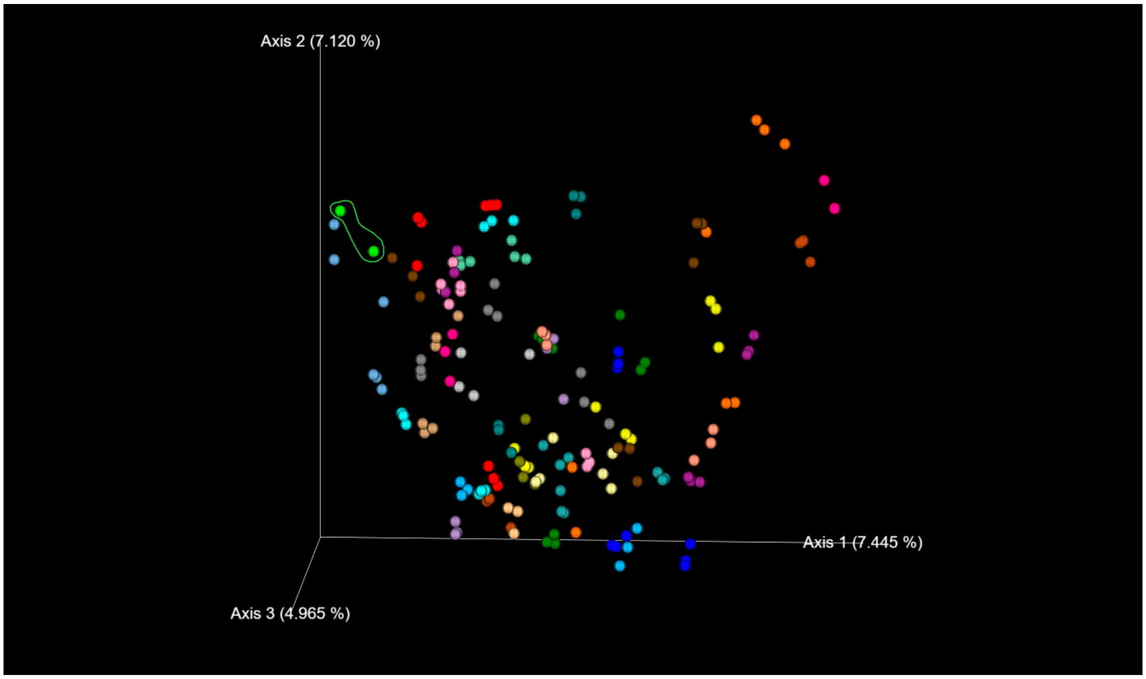

Supplement: Supplementary file 1 [file biomedicines-14-02123-s001.zip › Supplementary Figure S1. Technical replicate concordance (Emperor plot).pdf]
